# Integrated evaluation of workplace exposures and biomarkers of bladder cancer among textile dyeing workers

**DOI:** 10.1186/s42506-024-00167-7

**Published:** 2024-09-17

**Authors:** Amal Saad-Hussein, Safia Beshir, Weam Shaheen, Inas A. Saleh, Mohamed Elhamshary, Atef M. F. Mohammed

**Affiliations:** 1 Environmental & Occupational Medicine Department, Environment and Climate Change Research Institute, National Research Centre, Giza, Egypt; 2 Air Pollution Department, Environment & Climate Change Research Institute, National Research Centre, Giza, Egypt

**Keywords:** Textile dyeing, Polycyclic aromatic hydrocarbons, Volatile organic compounds, Bladder tumor Antigen, Nuclear matrix protein 22, 25-hydroxy vitamin D

## Abstract

**Background:**

The textile industry is the second risk factor for bladder cancer, after smoking. Previous studies focused on the impact of exposure to high concentrations of bladder carcinogenic chemicals in the textile dyeing industry on the elevation of bladder cancer biomarkers. This study aimed to evaluate bladder carcinogenic air pollutants in a textile dyeing factory and investigate its role and the role of serum 25-hydroxyvitamin D (25-OH vit. D) on cancer bladder biomarkers in exposed workers.

**Methods:**

A cross-sectional study was conducted. Particulate and vapor forms of polycyclic aromatic hydrocarbons (PAHs) and volatile organic compounds (VOCs) were monitored in the printing, dyeing, and preparing sections of a textile factory. Bladder tumor antigen (BTA), nuclear matrix protein 22 (NMP-22), and 25-OH vit. D were estimated in all the exposed workers (147 exposed workers) and in workers not occupationally exposed to chemicals (130 unexposed workers).

**Results:**

Aromatic bladder carcinogenic compounds were either in low concentrations or not detected in the air samples of working areas. BTA and NMP-22 of exposed workers were not significantly different from the unexposed. However, 25-OH vit. D was significantly lower in the exposed than unexposed workers. There was a significant inverse correlation between 25-OH vit. D and duration of exposure in exposed workers.

**Conclusion:**

The mean levels of PAHs and VOCs were within the safe standard levels in the working areas. The non-significant difference in BTA and NMP-22 between the exposed and unexposed groups suggests the presence of occupational exposures to safe levels of bladder carcinogenic aromatics, while the significantly lower 25-OH vit. D levels among the exposed than the unexposed groups could suggest the potential association of 25-OH vit. D with occupational exposures to low levels of PAHs and VOCs, and this association was found to be inversely correlated with the duration of exposures. Accordingly, more specific predictor tests must be applied for early diagnosis of bladder cancer among the exposed workers.

## Introduction

In the textile industry, the main sources of chemical hazards are the release from storage tanks, boilers, ovens, solvent-based diffusive sources, warehouses, and spills, as well as from the combustion of diesel engines and generators [1]. The textile industry includes several processes, starting from the manufacture of textile fibers to the production of fabric sheets, which are then sent to sales after the dyeing of the prepared fabrics.

Workplace exposure to chemical hazards created by textile dyeing processing is hazardous to the health of the surrounding workers. Organic releases, such as polycyclic aromatic hydrocarbons (PAHs), are produced from the textile materials containing lubricating oils and plasticizers when subjected to heat, that can volatilize or be thermally degraded turning these substances into volatile substances [1, 2].

Since the last century, the second important risk factor for the development of bladder cancer is occupational exposure in the textile industry, after the risk of tobacco smoking, the relative risk (RR) of occupational exposures was 13.4 [95% CI 1.5–48.2] in dye workers [3]. van Hoogstraten, et al. [4] found that 20% of all bladder cancers could be attributed to occupational exposures to aromatic compounds, such as benzidine, 4-aminobiphenyl, b-naphthylamine, 4-chloro-o-toluidine, as well as polycyclic aromatic hydrocarbons (PAHs) in the textile dye industry. These chemical exposures could be specific agents associated with bladder cancer [5]. Apart from aromatic amines, exposure to other toxic compounds present in the textile workplace in the form of industrial oils/cutting fluids, metals, dyes, paints, chlorinated hydrocarbons, and other solvents was found to be an increasing risk factor for bladder cancer [6]. These exposures may promote toxicity, and inform of mutagenic and carcinogenic effects to the exposed workers [7].

The main circulating form of vitamin D in the human body is 25-OH vit. D. Previous studies detected the inhibitory role of vitamin D on the proliferation and induction of apoptosis in vitro in human bladder tumor cells, which may consider its effect as a potential therapeutic on bladder malignancy [8, 9].

This study aimed to monitor the bladder carcinogenic air pollutants in a textile dyeing factory and investigate the relationship between 25-OH vit. D and cancer bladder biomarkers in workers exposed to PAHs in textile dyeing processes.

## Methods

### Industrial dyeing processes in the included factory

The textile dyeing processes in the selected factory are done in three steps, as observed during the walk-through survey. Each step is done in a special working section, but all are in the same open-air working place. The three steps are processing (preparation: of the fabric and select the appropriate dyes for each fabric type), dyeing (soaking the prepared fabrics in the dyes), and printing (finishing step, in which undesirable impurities are removed from the materials and print the required shapes).

### Study design and sample size

A cross-sectional study was conducted between March 2022 to February 2023. All the workers in the three textile dyeing sections (n=147) were included in the study (Exposed group), and 130 unexposed workers were included. The unexposed workers were chosen from the workers in a wastewater treatment plant not occupationally exposed to dyes during their working day, The two groups of the exposed and unexposed workers were matched in their age, socioeconomic levels, and educational levels, and both worked in open-air working areas. After obtaining written consent from the included individuals, all were subjected to the questionnaire, clinical examination, and blood sampling.

#### The inclusion criteria

- Exposed workers occupationally exposed to textile dyeing processes for more than 5 years, and their ages between 25 and 50 years.
- Unexposed subjects were not occupationally exposed to chemicals used in textile dyeing processes and matched in age range and socioeconomic status of the exposed workers.
- The exposed and unexposed workers must be working during the day shift, and they must not take any vitamins in the last 6 months, especially vitamin D.

#### Exclusion criteria

- Workers with previous history of exposure to chemicals used in textile dyeing were excluded from the unexposed workers

### Environmental assessments

Environmental monitoring was done in an Egyptian textile factory. Polycyclic aromatic hydrocarbons (PAHs) in their particulates and vapor forms and volatile organic compounds (VOCs) samples were collected bi-weekly from three areas (printing area, processing area, and dyeing area). From the printing area, 30 samples from five sites, including the printing room, drawing and design office, printing machine site, roasting site, and customer reception desk. From the processing area, 30 samples from six sites, including ram-1, ram-2, and ram-3,4 (at the front and the end of the hall). Furthermore, from the dyeing area, 28 samples from 4 sites, including laundry 1 at the front of the hall (closed machine), laundry 1 at the end of the hall (open machine), laundry 2 at the front of the hall (open machine), and laundry 2 at the end of the hall (closed machine).

#### Polycyclic aromatic hydrocarbons (PAHs)

Polycyclic aromatic hydrocarbons (PAHs) in the textile factory were collected on Whatman grade GF/A glass microfiber filters [10–13], using the low volume sampler technique (The ARA N-FRM Sampler and BOECO Vacuum Pump R-300) with an average flow rate of 10–12 l/min. Firstly, the filters were impregnated in acetone for 24 h to remove all organic contamination, and then heated at 400 °C for four hours. The processed filters were saved in desiccators till used for sampling. After collecting suspended particulate matter (SPM) samples, sample filters were covered with aluminum foil and stored for 24 h in desiccators in darkness. SPM filters were weighed till obtained constant weight to estimate SPM concentration. SPM samples were used for PAHs analysis; each filter was transferred to a glass flask. Ultrasonic bath and DCM/n-hexane (10 ml of 1:1, v:v) were used for extraction of PAHs, three times and for 10 min at room temperature. A column filled with 10 gm silica gel (70–230 mesh, ASTM purchased from Merck) and 2.0 gm anhydrous sodium sulfate was used for extraction cleanup. A rotary evaporator was used to concentrate the obtained extraction. The gas chromatography technique (limit of detection ranged from 0.50 to 3.91 ng/m^3^) was used to identify and evaluate the levels of the 16 EPA PAHs, which were expressed in ng/m^3^. Standard solution mixture of 16 PAH compounds (Supelco, Inc., Bellefonte, PA, 2000 μg/ml for each PAH) [14].

#### Classification of PAHs according to molecular weight

In this study, the method developed by Patel et al. [15] was used for the classification of PAHs as low molecular weight (LMW) or 2 rings, which were more water-soluble, moderate molecular weight (MMW) or 3 and 4 rings, which were moderate water-soluble, and high molecular weight (HMW) or 5 and 6 rings, which were less water-soluble.

#### Estimation of VOCs in the workplaces

The NIOSH standard method number 2549 was used for sampling and analysis of VOCs from the work environment. In this method, activated charcoal tube type ORBOTM-32 activated coconut charcoal (20/40) was used for VOCs air sampling according to NIOSH, 1996 [16]. Atmospheric air was drawn through tubes, by using a calibrated vacuum pump with a flow rate of 0.2 l/min (low volume sampler method: The ARA N-FRM Sampler and BOECO Vacuum Pump R-300). Sample tubes were stored in special plastic bags and kept in a freezer till processed not more than 15 days. Carbon disulfide (CS_2_) was used for VOCs extraction. 2.0 ml of CS2 was added to the loaded charcoal, and then a mechanical shaker was used for shaking. It stood for at least one hour to obtain the final solution. A Gas chromatography technique (limit of detection ranged from 0.001 to 0.02 mg/m^3^) was used for identifying and evaluating the concentrations of the individual VOCs [16].

### Tools

#### Questionnaire

Personal interviews were conducted with all the included subjects to fulfill personal, history, family history of cancers, and medical questionnaire, including bladder cancer symptoms (in the form of unpainful hematuria occurring suddenly, problems emptying the bladder, false sensation of a full bladder, burning feeling when passing urine, pain while urinating, lower abdominal or back pain), [17]. The questionnaire also thoroughly asked about exposure to environmental and occupational pollutants: hours of working per day, use of personal protective equipment (PPE), hours of exposure to sunlight, any drug taken, or vitamins, especially vitamin D.

#### Clinical examination

An occupational medicine specialist conducted abdominal-pelvic medical examinations for all the included persons to detect if there was any swelling, masses, or stiffness [17, 18], but per rectum examination was not done for the workers, as all of the included workers refused that.

#### Blood sampling and analysis

A 5.0-ml venous blood was obtained from each participant. A portion of the blood was placed in a clean tube and centrifuged to separate the serum. The tumor biomarkers bladder tumor antigen (BTA), nuclear matrix protein 22 (NMP-22), and the serum 25-hydroxy vitamin D were measured by quantitative sandwich enzyme immunoassay technique (ELISA) using ELISA kit from SinoGeneClon Biotech Co., Ltd. The specimens required for this kit are serum, plasma, and other biological fluids (SinoGeneClon Biotech www.sinogeneclon.com). In the present work, the serum of the included subjects was used.

The concentration of BTA in the samples was determined by comparing the O.D. of the samples to the standard curve (https://www.sinogeneclon.com/plus/view.php?aid=690).

The concentration of NMP-22 in the samples was determined by comparing the O.D. of the samples to the standard curve (https://www.sinogeneclon.com/plus/view.php?aid=4447).

Serum 25-hydroxy vitamin D (25-OH vitamin D) was measured by ELISA kit from EDI Epitope Diagnostics, Inc. The kit is designed, developed, and produced for the quantitative measurement of total 25-OH vitamin D in serum utilizing the competitive immunoassay technique. This assay utilizes a monoclonal antibody that binds to both 25-OH vitamin D2 and 25-OH vitamin D3 equally.

The concentration of a total 25-OH vitamin D in samples was determined directly from this calibration curve (https://www.sinogeneclon.com/plus/view.php?aid=2414).

### Statistical analysis

The collected data were statistically analyzed using the SPSS package for Windows version 23. The quantitative data was presented as mean ± standard deviation (SD).

The limit of detection (LOD) was calculated based on the standard deviation (σ) of PAHs concentrations and the slope of the calibration curve (s) at levels approximating the LOD according to the formula [19]:

$$\text{LOD }= 3.3(\upsigma /\text{S}),$$

The smoking index (SI) was calculated for each smoker in both exposed and unexposed groups according to the number of cigarettes smoked per day (CPD) and number of years of tobacco use through the following formula [20]:

$$\text{SI }=\text{ CPD }\times \text{ years of tobacco use}$$

The comparisons were done first between the exposed and the unexposed workers using the independent *t*-test, and then, between the exposed workers according to their different tasks using Analysis of variance (ANOVA) and least significant differences (LSD) as a post-hoc test. Pearson’s correlation coefficient was used to test the relationships of the results. The level of significance was at *P* value < 0.05.

## Results

The sum of the average concentrations of PAHs in the air of the work environment was the highest at the printing (391.2 ng/m^3^), followed by the processing (334.7 ng/m^3^) and the dyeing (73.6 ng/m^3^) areas, without significant differences between the values of the individual contents of particulate and the average air levels of ΣPAHs in the wastewater treatment plant was 56.1 ng/m^3^. PAHs between the three included working areas (printing, processing, and dyeing area). Even Indeno (1,2,3-c,d) pyrene was higher in the dyeing area compared to the printing and processing areas, and in the processing area compared to the printing area, but not to the level of significance (*p* = 0.051). There is no TLV-TWA for PAHs compounds, except for Naphthalene, which was lower in the three areas than the TLV-TWA level (Table 1).

**Table 1:** The mean concentrations of high molecular weight particulate polycyclic aromatic hydrocarbons (PAHs) in the air of the work environment in the textile dyeing factory

Polycyclic aromatic hydrocarbons (PAHs)		Printing area (*N* = 30)	Processing area (*N* = 30)	Dyeing area (*N* = 28)	LOD	TLV-TWA^a^ (ng/m^3^)	*P* value
Naphthalene	NAP-2	38.8	45.4	15.0	0.82	52.0 × 10^6^	0.518
Acenaphthylene	ACY-3	15.5	31.9	7.5	0.48	0.2 × 10^6^	0.327
Acenaphthene	ACE-3	27.7	24.7	6.6	0.71	0.2 × 10^6^	0.499
Phenanthrene	PHE-3	29.3	24.9	5.4	0.53	0.2 × 10^6^	0.188
Fluorene	FLU-3	35.5	19.3	4.2	0.92	0.2 × 10^6^	0.366
Anthracene	ANT-3	8.9	22.0	2.7	0.50	0.2 × 10^6^	0.150
Fluoranthene	FLT-4	7.7	12.0	2.3	0.58	0.2 × 10^6^	0.364
Pyrene	PYR-4	123.7	18.5	2.9	3.91	0.2 × 10^6^	0.129
Benzo(a)anthracene	BAA-4	14.3	7.6	2.3	1.07		0.211
Chrysene	CRY-4	21.7	10.4	4.3	1.52		0.404
Benzo(b)fluoranthene	BBF-5	3.0	11.3	1.1	0.48		0.106
Benzo(k)fluoranthene	BKF-5	11.4	10.1	2.8	0.83		0.554
Benzo(a) pyrene	BAP-5	8.6	9.8	1.8	0.56		0.359
Dibenzo(a,h)anthracene	DBA-5	4.6	7.1	2.2	0.59		0.312
Indeno(1,2,3-c,d)pyrene	IND-6	10.9	37.0	4.9	0.58		0.051
Benzo(ghi)perylene	BGP-6	29.7	41.9	7.5	0.50		0.108
**ΣPAHs**		**391.2**	**334.7**	**73.6**	**–**		

*LOD* limits of detection, *TLV-TWA*^a^ cited from ACGIH website: https://www.acgih.org/naphthalene/

Table 2 shows the relative distribution of PAHs according to molecular weight in the textile dyeing factory. The higher contribution percentages % of PAHs were MMW (72.6% in the printing area, 51.2% in the processing area, and 52.0% in the dyeing area), which indicated that the sources of the individual PAH concentrations were mainly from combustion and heating activities (Table 2).

**Table 2:** Relative distribution (%) of PAHs according to molecular weight in the textile dyeing factory

%	Printing area (%)	Processing area (%)	Dyeing area (%)
LMW (2 rings)	9.9	13.6	20.4
MMW (3 rings + 4 rings)	72.6	51.2	52.0
HMW (5 rings + 6 rings)	17.5	35.2	27.6

*PAHs* polycyclic aromatic hydrocarbons, *LMW* low molecular weight, *MMW* moderate molecular weight, *HMW* high molecular weight

The individual VOCs released from the textile dyeing industrial processes are presented in Table 3. The concentrations of the individual VOCs were below the VOCs Egyptian threshold limit values-time weighted average (TLV-TWA). The individual VOCs (chloroform and p-xylene) were detected in the air samples in the three areas but were below the Egyptian TLV-TWA [21]. Chloroform was detected in the upper international Threshold limit values (TLV) (10 mg/m^3^) according to the American Conference of Governmental Industrial Hygienists (ACGIH) [22]. Acetic acid, dimethyl formamide, trichloro ethylene, benzaldehyde, and benzene acetaldehyde were significantly higher in the dyeing areas compared to the printing and processing areas. Moreover, the concentrations of the aromatic compounds carcinogenic to the bladder, such as benzidine, 4-aminobiphenyl, b-naphthylamine, and 4-chloro-o-toluidine, were not detected in any of the samples collected from the three areas (printing, processing, and dyeing section areas) (Table 3).

**Table 3:** The average concentrations of individual VOCs emitted from the textile dyeing industry

VOCs	Concentration (mg/m^3^)					*P* value	LOD
	Printing area (*N* = 30 samples)	Processing area (*N* = 30 samples)	Dyeing area (*N* = 28 samples)	VOCs Egyptian acceptable limit (21) (mg/m^3^)	TLV ACGIH (22) (mg/m^3^)		
Ethanol	1.83	5.41	6.23	1880	1884	.132	0.01
Chloroform	3.66	5.94	10.72	49	10	.150	0.01
Formaldehyde	0.06	0.15	0.08	0.37	0.37	.099	0.004
Isopropanol	0.63	0.94	1.08	983	492	.612	0.02
p-Xylene	2.50	0.71	0.98	434	434	.524	0.01
Carbon tetrachloride	0.09^a,b^	0.46^c^	1.05	31	31	.000	0.01
Chloro benzene	0.62	0.70	0.98	46	46	.716	0.004
Ethylbenzene	1.12	0.38	1.14	434	434	.330	0.004
Benzene	1.56	0.47	1.23	1.6	1.6	.255	0.02
Phenol	0.28	0.58	1.04	19	19	.05	0.01
Propanoic acid	0.28	0.59	0.60	1.5	30	.390	0.01
Styrene	0.83	0.71	0.96	40	85	.861	0.007
Toluene	0.01	0.01	0.02	0.036	75	.796	0.001
Acetaldehyde	1.19	0.72	0.64	45.75	45	.647	0.02
Acetic acid	1.90^b^	3.36	6.28	25	25	.027	0.01
Dimethyl formamide	0.15^b^	0.45^c^	1.09	30	30	.000	0.01
Ethylene glycol	0.61	0.53	1.03	100	63	.252	0.01
Pyridine	0.61	0.79	0.44	16	16	.570	0.003
Trichloro ethylene	0.57^b^	0.28^c^	1.34	269	134	.001	0.01
Benzaldehyde	0.24^b^	0.42^c^	1.06	–	0.22	.026	0.01
Benzene acetaldehyde	0.23^b^	0.60	1.16	–	–	.024	0.01
Pregnene	0.14	0.54	0.44	–	1180	.231	0.01
Acetyl aspartyl glutamic acid	0.24	0.69	0.76	–	–	.216	0.02
Phenoxyethanol	1.24	3.68	4.41	–	0.17	.237	0.01
Benzidine	N.D	N.D	N.D	–	–		
4-aminobiphenyl	N.D	N.D	N.D	–	–		
b-naphthylamine	N.D	N.D	N.D	–	–		
4-chloro-o-toluidine	N.D	N.D	N.D	–	–		

*N* no. of air samples, *LOD* limits of detection, *N.D.* not detected through gas chromatography technique, *TLV* threshold limit values, *ACGIH* American Conference of Governmental Industrial Hygienists

^a^Significant difference between printing and processing

^b^Significant difference between printing and dyeing

^c^Significant difference between processing and dyeing

All the included participants of the exposed and unexposed workers were males. The average years of employment of the exposed workers were 15.2 ± 7.7 years, and the average air levels of ΣPAHs and TVOCs in the working area of the unexposed workers were 56.1 ng/m^3^ and 17.1 mg/m^3^ respectively. According to the questionnaire, there were no symptoms of bladder cancer in the exposed and unexposed workers. Clinical examination for all the examined workers revealed that their abdominal shape was normal, and there were no cases of abdominal stiffens and no masses were felt.

The comparisons of the age, smoking index (SI), bladder cancer tumor biomarkers, and 25-OH vit. D between the exposed and unexposed groups showed that there was no significant difference in the age and smoking index (SI) between the exposed and unexposed workers. BTA and NMP-22 were slightly higher in the exposed workers compared to their unexposed but without significant difference. While 25-OH vit. D was significantly higher in the exposed workers compared to the unexposed workers (Table 4).

**Table 4:** Comparisons of the tumor biomarkers and vitamin D levels between the exposed workers and unexposed workers

Tumor biomarkers and vitamin D levels	Exposed workers (*N* = 147)		Unexposed (*N* = 130)		*P* value
	Mean	SD	Mean	SD	
Age (years)	37	0.6	40.9	9.7	*P* > 0.05
SI (cig. years)	3.2	1.1	3.3	0.6	*P* > 0.05
BTA ng/ml	1.71	0.17	1.42	0.09	*P* > 0.05
NHP-22 ng/ml	12.55	0.48	11.78	1.53	*P* > 0.05
25-OH vit. D ng/ml	23.92	0.48	39.85	1.55	≤ 0.0001

*SI* smoking index

**Fig. 1:**
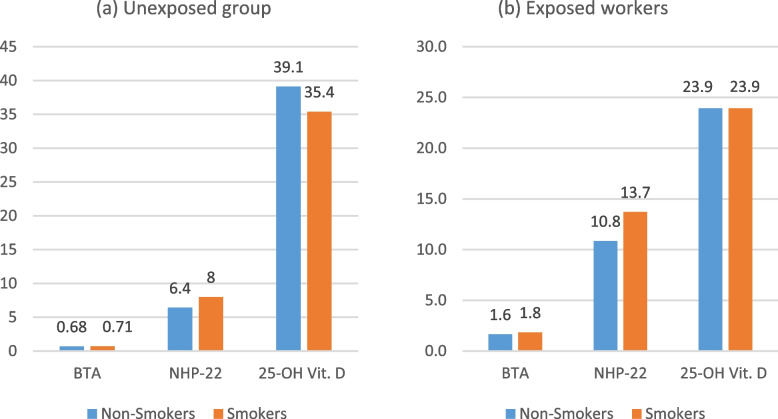
Comparison of the BTA, NMP-22, and 25-OH vit. D between smokers and non-smokers in the unexposed (*N* = 130) and exposed workers (*N* = 147)

The levels BTA, NMP-22, and 25-OH vit. D of the unexposed workers were not significantly different in the smokers compared to the non-smokers (*p* = 0.862, *p* = 0.131, and *p* = 0.615 respectively), (Fig. 1a), and of the exposed workers were not significantly different in the smokers compared to the non-smokers (*p* = 0.608, *p* = 0.383, and *p* = 0.993 respectively), (Fig. 1b).

The 25-OH vit. D was significantly the lowest in printing workers (21.5 ng/ml) compared to dyeing (24.1 ng/ml) and processing workers (28.4 ng/ml), respectively, and was significantly higher in processing workers compared to the dyeing workers. There were no significant differences in BTA and NMP-22 between the workers in the three working areas (Table 5).

**Table 5:** Comparisons of the tumor biomarkers and vitamin D levels between the dyeing workers in the different departments

Tumor biomarkers and vitamin D levels	Printing (*N* = 39)		Processing (*N* = 18)		Dyeing (*N* = 90)		ANOVA
	Mean	SD	Mean	SD	Mean	SD	*P*-value
BTA ng/ml	1.50	0.48	2.33	0.18	1.69	0.19	*P* > 0.05
NMP-22 ng/ml	9.18	3.97	12.65	1.32	12.77	1.77	*P* > 0.05
25-OH vit. D ng/ml	21.5^**(a,b)**^	0.95	28.4^**(a,c)**^	1.32	24.1^**(b,c)**^	0.56	≤ 0.0001

According to LSD:

^a^Significant difference between printing and processing workers

^b^Significant difference between printing and dyeing workers

^c^Significant difference between processing and dyeing workers

There was a significant inverse correlation between 25-OH vit. D and the duration of exposure in the exposed workers, but there was no significant relationship with the tumor biomarkers. Also, there was no significant relationship between 25-OH vit. D and the tumor biomarkers (Table 6).

**Table 6:** Relationship between duration of exposure, 25-OH vit. D and the tumor biomarkers levels in the exposed workers (*n* = 147)

			Duration of exposure (years)	25-OH vit. D ng/ml
Exposed workers	25-OH vit. D ng/ml	*r*	− 0.3^*^	1
		*P* value	< 0.05	
	BTA ng/ml	*r*	0.04	0.07
		*P* value	> 0.05	> 0.05
	NHP-22 ng/ml	*r*	− 0.01	0.02
		*P* value	> 0.05	> 0.05

Pearson’s correlation coefficient was used

^*^P value < 0.05

## Discussion

There is great evidence that occupational exposure to aromatic amines and PAHs increases the development of urothelial bladder malignancy and is considered an important risk factor [23]. The presence of PAHs in the working environment may be attributed to dyes, acids, bleaching powder, metal ions, and other chemicals used for printing, processing, and dying textiles [24]. In addition, these can be caused by solvents and kerosene, which release pollutants during printing into the air [8].

Low-temperature combustion processes result in LMW-PAHs pollutants, while, high-temperature combustion processes originate in MMW-PAHs and HMW-PAHs pollutants [25]. The incomplete combustion was considered to be the main source of PAH releases in various industrial activities including textile factories [15].

In the present study, the overall average concentrations of PAHs in the textile dyeing factory were the highest in the printing area, followed by the processing and dyeing areas. The relative distribution of PAHs according to molecular weight showed that the highest contribution percent of PAHs was MMW and was detected in the printing area. This means that the increase in the PAHs concentrations in the printing area was attributed mainly to the high-temperature combustion used for heating activities done in this area whereas LMW-PAHs were detected in higher concentrations in the dyeing area, as only a low heating process was done in this area.

Occupational exposure to aromatic amines (2-naphthylamine, 4-aminobiphenyl, and benzidine) and 4,4′-methylenebis (2-chloroaniline), are considered to be the main risky pollutants for the development of bladder cancer in many chemical industries, including textile dyeing industry [26]. There is strong evidence of a causal link between bladder cancer and these compounds based primarily on a Group 1 designation by the International Agency for Research on Cancer (IARC). Through the last updated list of the classifications of carcinogenic agents by cancer sites (2023), IARC mentioned these compounds among the bladder carcinogenic agents with sufficient evidence in humans.

In the present study, these risky chemicals were not detected in all the air samples collected from the three areas of the selected textile dyeing factory, and all the other PAH and VOCs were under the National and International TLN-TWA under the TLVs of ACGIH. This could be attributed to exposure to low concentrations of the air pollutants detected in the workplace, in addition, all the dyeing procedures in the present study were found to be done in open-air working areas. These undetected levels of the bladder carcinogenic chemicals in the working environment of the selected factory in this study could explain, the non-significant difference between the exposed and unexposed workers in the bladder tumor biomarkers BTA and NMP-22. These two bladder cancer biomarkers were also non-significantly different between the workers in the different areas of the included factory.

The International Labor Organization (ILO) always recommends applying environmental workplace regulations in the form of substitution of these toxic materials for others that are non-toxic, as well as adequate ventilation, proper usage of local exhaust ventilation, and limitation of the occupational exposure time as these regulations were found also to decrease the incidence of cancers [27]. Therefore, in the present study, the low concentrations of PAHs in the workplace of the textile dyeing workers because the working areas were open areas, could be one of the reasons for the non-elevation of bladder cancer tumor biomarkers compared to the unexposed group.

A meta-analysis study revealed that vitamin D deficiency is associated with an increase in the risk of bladder malignancy [28]. Occupational exposures to VOCs were found to be associated with a decrease in serum vitamin D levels [29]. They found that elevation of the chloroform and m-/p-xylene (individual VOCs) could have a significant effect on vitamin D levels. Liu et al. [29] suggested that there is an interaction effect between these individual VOC exposures and the serum vitamin D in the exposed workers.

Serum levels of 25-OH vit. D measures vitamin D status in the human body whatever its sources are from sunlight exposure, diet, or therapeutic supplementations [30]. Moreover, through a systemic review, several studies detected protective effects of high serum vitamin D levels against the development of bladder malignancy [31].

In the present study according to the reference of the kit used, the recommended sufficient level of 25-OH vit. D is to be more than 30 ng/ml (i.e., those at the level 30 ng/ml do not need supplementation or therapy), insufficiency level is 21–29 ng/ml, and deficiency level is less than 20 ng/ml. Therefore, the 25-OH vit. D was significantly insufficient in the exposed workers (23.92 ± 0.48 ng/ml) compared to the unexposed workers (39.85 ± 1.55 ng/ml). Besides, there was a significant inverse correlation of 25-OH vit. D with the duration of occupational exposures. This could be attributed to the detection of the individual VOCs chloroform and p-xylene in the working place of the selected factory.

In the present study, the average concentration of chloroform was found to be at the high TLVs of ACGIH in the dyeing area and lower in the other two areas but was lower than the Egyptian accepted limits. Moreover, the average concentration of p-xylene was found to be the highest in the printing area and lowest in the processing area but was lower than the Egyptian limits and under the TLVs of ACGIH in the three areas. The associations between vitamin D levels and the exposure concentrations to chloroform and m-/p-xylene were found to be U-shaped [29]. Thus, this may explain the present results of 25-OH vit. D in the exposed workers. The average serum level of 25-OH vit. D was significantly lower in the printing and dyeing workers compared to that in processing workers, and that was true with the low concentrations of p-Xylene in the processing area than in the other two areas, and of chloroform in the processing area compared to the dyeing area. It was also found that the average serum level of the 25-OH vit. D was significantly lower in the printing workers compared to the dyeing workers which could be attributed to the high average concentration of p-Xylene in the printing area compared to the dyeing area.

Moreover, a significant inverse correlation was found between particulate PAH levels and vitamin D [32]. The results of Chen et al.’s study [32] indicated a significant negative dose–response relationship between vitamin D deficiency and the increase of OH-PAH levels. In the current study, the average concentrations of PAHs in the workplace air in the textile dyeing factory were arranged in the order Printing area > Processing area > Dyeing area. This could explain the significant effect on 25-OH vit. D in the exposed workers, as the level of 25-OH vit. D was significantly insufficient in the printing workers (21.5 ± 0.95 ng/ml) compared to those in the other two exposed groups. Therefore, the decline in 25-OH vit. D levels in the printing workers could be attributed to their occupation exposure to the elevation in the average concentrations of PAHs and the individual VOCs p-xylene in their working area. The decline in 25-OH vit. D levels in the dyeing workers could be attributed to their exposure to the individual VOCs p-xylene and chloroform in their working area.

Previous studies have detected an association between vitamin D deficiency and the increased risk of lung cancer [33] and bladder carcinoma [28]. Moreover, several epidemiological studies detected a preventive beneficial role of vitamin D in cancers, such as colorectal and breast cancers [30, 34]. This could be due to the involvement of vitamin D in the cellular pathways that lead to cancer prevention [35, 36]. Moreover, Feldman et al. [35] denoted the antitumor activities of 25-OH vit. D through mediating cell differentiation and apoptosis, and inhibition of angiogenesis and metastasis. In vitro, vitamin D was found to inhibit proliferation and induce apoptosis in human bladder tumor cells, therefore, it may have a potential therapeutic effect on bladder cancer [9, 37].

In the present study, there was no association between the levels of 25-OH vit. D and the bladder cancer tumor biomarkers, which could be due to the non-significant change of these tumor biomarkers of the included workers compared to the unexposed group, or even between the workers in the different working areas.

### Limitations and strengths of the study

The main limitation was to find workers who were not occupationally exposed to PAHs and VOCs and matched with the textile workers, so workers from a wastewater treatment plant not occupationally exposed to these chemicals were selected to be the unexposed workers.

The second limitation is that all the included workers in the two groups refused to be examined per-rectum, so clinical examination was limited to detecting any swelling and the general condition of the workers.

The strength was the novelty of the idea of the study, as most of the published papers discuss the relation between exposure to high levels of bladder carcinogenic exposures and the elevation of bladder tumor biomarkers, but this study investigated the relation between the undetectable concentrations of bladder carcinogenic chemicals in the air of the working places and the levels of bladder tumor biomarkers.

## Conclusion

The mean levels of PAHs and VOCs were within the safe standard levels in the working areas. The non-significant difference in BTA and NMP-22 between the exposed and unexposed groups suggests the presence of occupational exposures within the safe levels of the bladder carcinogenic aromatics, while the significantly lower 25-OH vit. D levels among the exposed than the unexposed groups could suggest the potential association of 25-OH vit. D with occupational exposures to low levels of PAHs and VOCs, and this association was found to be inversely correlated with the duration of exposures. Accordingly, more specific predictor tests must be applied for early diagnosis of bladder cancer among the exposed workers.

Therefore, it was recommended that textile dyeing processes be done in open working areas to limit occupational exposures to high bladder carcinogenic particulate PAHs and VOCs. This change could significantly reduce the levels of bladder cancer biomarkers in textile dyeing workers. In addition, improving vitamin D status through different sources, such as sunlight exposure, dietary habits, or therapeutic supplementations, could play an important role in preventing the elevation of bladder cancer biomarkers.

## Data Availability

The original data is available when requested.
